# Dynamic changes in AI-based analysis of endometrial cellular composition: Analysis of PCOS and RIF endometrium

**DOI:** 10.1016/j.jpi.2024.100364

**Published:** 2024-02-01

**Authors:** Seungbaek Lee, Riikka K. Arffman, Elina K. Komsi, Outi Lindgren, Janette Kemppainen, Keiu Kask, Merli Saare, Andres Salumets, Terhi T. Piltonen

**Affiliations:** a Department of Obstetrics and Gynaecology, Research Unit of Clinical Medicine, Medical Research Center, Oulu University Hospital, University of Oulu, Oulu 90220, Finland; b Department of Obstetrics and Gynaecology, Institute of Clinical Medicine, University of Tartu, Tartu 50406, Estonia; c Department of Pathology, Oulu University Hospital, Cancer and Translational Medicine Research Unit, University of Oulu, Oulu 90220, Finland; d Competence Centre on Health Technologies, Tartu 51014, Estonia; e Division of Obstetrics and Gynaecology, Department of Clinical Science, Intervention and Technology, Karolinska Institute and Karolinska University Hospital, Stockholm 14152, Sweden

**Keywords:** Artificial intelligence, Endometrium, Polycystic ovary syndrome, Recurrent implantation failure, IVF, Computational histology

## Abstract

**Background:**

The human endometrium undergoes a monthly cycle of tissue growth and degeneration. During the mid-secretory phase, the endometrium establishes an optimal niche for embryo implantation by regulating cellular composition (e.g., epithelial and stromal cells) and differentiation. Impaired endometrial development observed in conditions such as polycystic ovary syndrome (PCOS) and recurrent implantation failure (RIF) contributes to infertility. Surprisingly, despite the importance of the endometrial lining properly developing prior to pregnancy, precise measures of endometrial cellular composition in these two infertility-associated conditions are entirely lacking. Additionally, current methods for measuring the epithelial and stromal area have limitations, including intra- and inter-observer variability and efficiency.

**Methods:**

We utilized a deep-learning artificial intelligence (AI) model, created on a cloud-based platform and developed in our previous study. The AI model underwent training to segment both areas populated by epithelial and stromal endometrial cells. During the training step, a total of 28.36 mm2 areas were annotated, comprising 2.56 mm2 of epithelium and 24.87 mm2 of stroma. Two experienced pathologists validated the performance of the AI model. 73 endometrial samples from healthy control women were included in the sample set to establish cycle phase-dependent dynamics of the endometrial epithelial-to-stroma ratio from the proliferative (PE) to secretory (SE) phases. In addition, 91 samples from PCOS cases, accounting for the presence or absence of ovulation and representing all menstrual cycle phases, and 29 samples from RIF patients on day 5 after progesterone administration in the hormone replacement treatment cycle were also included and analyzed in terms of cellular composition.

**Results:**

Our AI model exhibited reliable and reproducible performance in delineating epithelial and stromal compartments, achieving an accuracy of 92.40% and 99.23%, respectively. Moreover, the performance of the AI model was comparable to the pathologists’ assessment, with F1 scores exceeding 82% for the epithelium and >96% for the stroma. Next, we compared the endometrial epithelial-to-stromal ratio during the menstrual cycle in women with PCOS and in relation to endometrial receptivity status in RIF patients. The ovulatory PCOS endometrium exhibited epithelial cell proportions similar to those of control and healthy women’s samples in every cycle phase, from the PE to the late SE, correlating with progesterone levels (control SE, r2 = 0.64, FDR < 0.001; PCOS SE, r2 = 0.52, FDR < 0.001). The mid-SE endometrium showed the highest epithelial percentage compared to both the early and late SE endometrium in both healthy women and PCOS patients. Anovulatory PCOS cases showed epithelial cellular fractions comparable to those of PCOS cases in the PE (Anovulatory, 14.54%; PCOS PE, 15.56%, p = 1.00). We did not observe significant differences in the epithelial-to-stroma ratio in the hormone-induced endometrium in RIF patients with different receptivity statuses.

**Conclusion:**

The AI model rapidly and accurately identifies endometrial histology features by calculating areas occupied by epithelial and stromal cells. The AI model demonstrates changes in epithelial cellular proportions according to the menstrual cycle phase and reveals no changes in epithelial cellular proportions based on PCOS and RIF conditions. In conclusion, the AI model can potentially improve endometrial histology assessment by accelerating the analysis of the cellular composition of the tissue and by ensuring maximal objectivity for research and clinical purposes.

## Graphical Abstract

**Figure:**
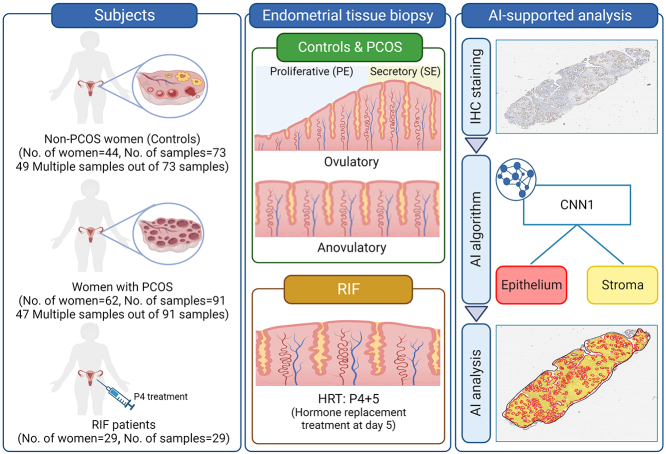

## Introduction

The epithelium and stroma are the main cellular compartments of the human endometrium, with their growth, differentiation, and shedding being timely and strictly regulated during the menstrual cycle. The core function of the endometrial lining is to coordinate embryo implantation and to support pregnancy until delivery.^1^ The uterine epithelium consists of the luminal epithelium (LE) and glandular epithelium (GE), with the LE having a core task in implantation by sensing the embryo and guiding it toward the correct embryonic orientation. On the other hand, the GE facilitates this process with glandular excretions, which are pivotal for endometrial receptivity, embryo nourishment, and embryo-endometrial crosstalk during implantation.^2^^,^^3^ The stromal compartment serves as a niche and nutritional source for the implanting embryo after it has passed the epithelial layer and invaded the endometrial stroma, as well as supporting the growth and survival of epithelial cells.^4^ Therefore, the crosstalk between the endometrial epithelium and the stroma and the correct cell-type ratio within the endometrium are essential for achieving successful implantation and a positive pregnancy outcome.^5^

In response to fluctuating levels of ovarian steroid hormones, the endometrial lining undergoes dynamic structural and functional changes throughout the menstrual cycle. During the proliferative phase (PE), estradiol (E2) stimulates cell growth and proliferation in both the epithelium and the stroma, leading to stromal expansion.^1^^,^^3^ Following ovulation, progesterone (P4) produced by the corpus luteum downregulates E2 receptors, inhibits cell mitoses, and promotes cell differentiation in both epithelial and stromal compartments, leading to stromal decidualization and receptive epithelial cells for implanting embryo.^6^ Due to the effects of P4 in the secretory phase (SE), the GE ceases growth and maturation and initiates protein excretion.^3^ This process is accompanied by a diminished stromal proportion in the endometrium.^1^ Therefore, adequate development of the epithelium and stroma and their ratio are essential for the window of implantation (WOI),^7^ and conversely, altered epithelium to stroma proportions during the WOI may lead to adverse reproductive outcomes, such as implantation failure and miscarriage.^7^, ^8^, ^9^

Infertility-associated conditions, like polycystic ovary syndrome (PCOS)^10^^,^^11^ and recurrent implantation failure (RIF),^12^ have been linked with endometrial dysfunction in the WOI. The endometrial dysfunction found in women with PCOS, a complex endocrine disorder affecting one in eight women, has been postulated as a potential contributor to subfertility in addition to irregular ovulation, as several studies have even suggested that the PCOS endometrium exhibits altered endometrial receptivity.^9^, ^10^, ^11^ Furthermore, women with PCOS have an elevated risk of endometrial hyperplasia^9^ and endometrial cancer,^9^, ^10^, ^11^ indicating excessive cell growth.

RIF is considered when a woman experiences three or more unsuccessful in vitro fertilization (IVF) attempts with good-quality embryo transfers.^7^ While previous studies have identified various risk factors (e.g., maternal age, chromosomal aberrations in the embryos, and disrupted endometrial maturation) for RIF, its exact underlying mechanisms remain obscure.^8^ Specifically, the assessment of endometrial development, crucial for a successful pregnancy in response to IVF treatment, including hormone replacement treatment (HRT), remains unexplored. In this case, an artificial intelligence (AI) tool analyzing the proportion of epithelial and stromal fractions could offer a new approach to evaluating the histological features of infertility-associated endometrium, such as PCOS and RIF.^13^

In the present study, we utilized an AI model introduced in our previous study^14^ to analyze a series of endometrial histology images for epithelial and stromal proportions. We examined a total of 193 endometrial tissues based on: (1) menstrual cycle phases, (2) ovulatory status in women with PCOS, and (3) HRT response in RIF patients. Here, we demonstrated excellent performance of the AI model in segmenting epithelial and stromal cell compartments in different endometrial conditions.

## Materials and methods

### Sample collection

#### PCOS samples and control samples

**Fig. 1:**
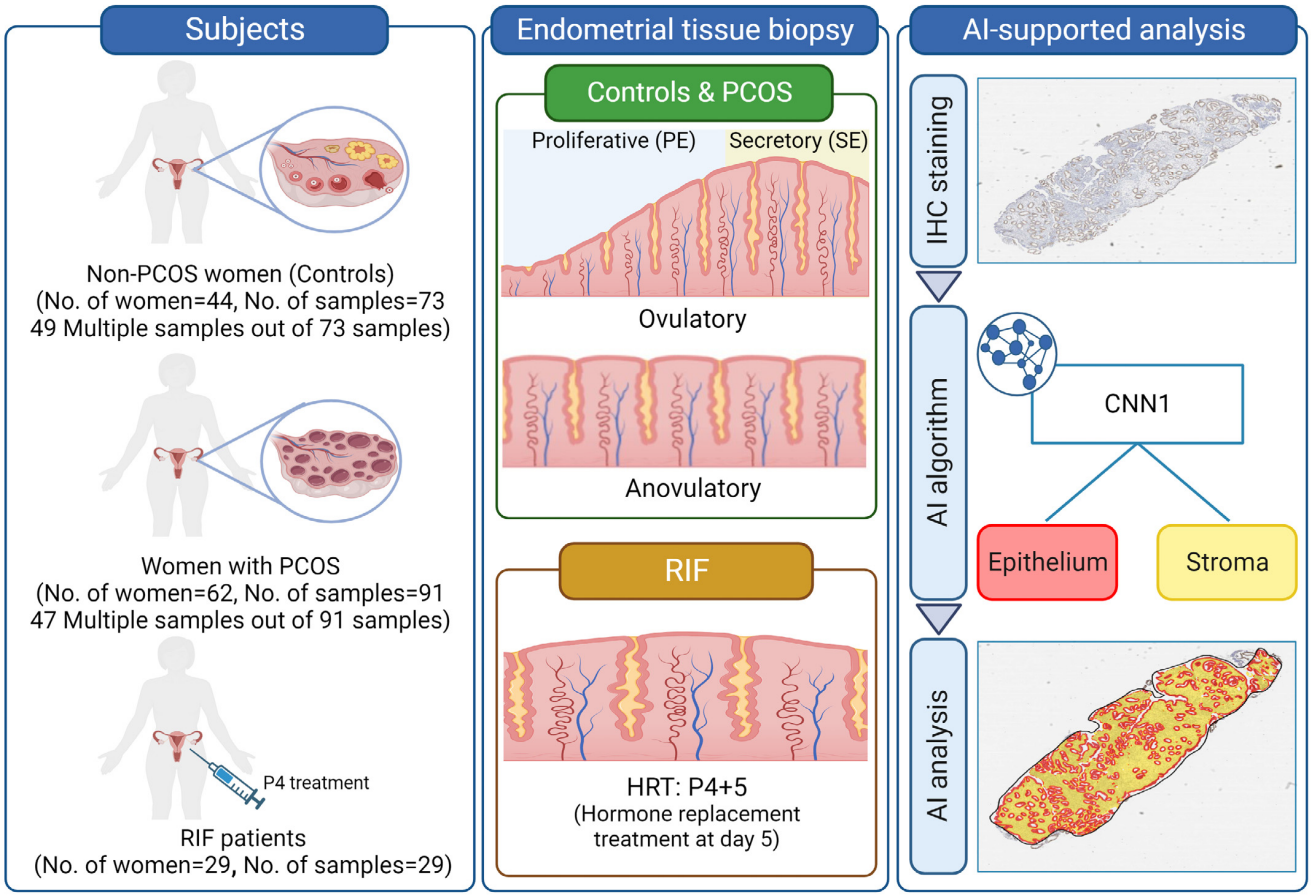
Graphical overview of sample information and the AI analysis protocol. Twenty (20) control women and 18 PCOS women provided multiple samples in different cycle phases during different menstrual cycles. The endometrial biopsy samples were categorized according to menstrual cycle phase, ovulation, and endometrial receptivity. The AI model was trained to segment the epithelium (red) and stroma (yellow) using IHC slides. PE, proliferative phase; SE, secretory phase; P4, progesterone; HRT, hormone replacement treatment; IHC, immunohistochemistry; CNN, convolutional neural network.

A total of 106 women were recruited at Oulu University Hospital (Jan 2017–Mar 2020), of whom 62 were diagnosed with PCOS according to the Rotterdam criteria (Fig. 1).^15^ The study was approved by the Regional Ethics Committee of the Northern Ostrobothnia Hospital District, Finland (65/2017), and an informed consent form was signed by all study subjects. The women with PCOS included anovulatory women as well as women with occasional ovulations that were traced with urine luteinizing hormone (LH) testing. All control women (*n*=44) had regular menstrual cycles. Women were excluded if they were smokers or had used hormonal medications 3 months prior to sampling. Some women provided multiple samples (multiple sample providers) in different cycle phases during different menstrual cycles, limited to one biopsy per cycle, and with a maximum of three samples per person. A suction curette (Pipelle®) was used for the endometrial biopsy, and the biopsy samples were treated with formaldehyde or RNAlater (Ambion, Austin, TX, USA). The presence of the corpus luteum was confirmed by transvaginal ultrasonography (TVUS) via Voluson E8 (GE Healthcare Technologies). The endometrial samples obtained from ovulatory cycles were grouped based on the cycle phases determined by experienced pathologists; the PE on cycle days 6–8 (control *n*=12, PCOS *n*=24), or the SE: the early secretory phase (ESE, LH + 2–4 days) (control *n*=15; PCOS *n*=15), mid-secretory phase (MSE, LH + 7–8 days) (control *n*=26; PCOS *n*=21), and late secretory phase (LSE, LH + 11–12 days) (control *n*=20; PCOS *n*=19). In addition, anovulatory samples were collected from women with PCOS (PCOS *n*=12). To avoid the potential influence of age and body mass index (BMI) on the analysis results, these two factors were matched between controls and women with PCOS (age: controls, mean 32.16; PCOS, mean 33.29, *p*=0.19; BMI: controls, mean 26.78; PCOS, mean 28.35, *p*=0.12).^14^

The clinical parameters, including both endometrial thickness and sex hormone-related factors [i.e., follicle-stimulating hormone, LH, anti-Müllerian hormone, P4, testosterone, and free androgen index (FAI)], were measured using specific methodologies: TVUS, the cobas e411analyzer, and an Agilent 1290 Rapid Resolution LC System, respectively, as previously detailed in Lee et al.^14^

#### RIF samples

A total of 29 endometrial biopsy samples were collected from the RIF patients who registered for the beREADY endometrial receptivity testing (CCHT, Tartu, Estonia). The study was approved by the Research Ethics Committee of the University of Tartu, Estonia (340T-12). All data on the RIF patients were anonymized, as requested for ethics approval from the University of Tartu. The samples were obtained on day 5 after the beginning of P4 treatment (P4+5) (Fig. 1). The beREADY test, which assesses endometrial receptivity based on receptivity-related gene expression,^16^^,^^17^ was performed on all 29 RIF patients.^14^ The endometrial RNA was extracted with a Qiagen miRNeasy Mini kit and sequenced and analyzed in CCHT. The samples were divided into three groups according to the receptivity test results: pre-receptive (*n*=9), receptive (*n*=10), and post-receptive (*n*=10).

Detailed study protocols and the baseline characteristics of all three study groups are available in our previous publication.^14^

### Immunohistochemistry (IHC)

We utilized the same IHC slide sets from our previous study.^14^ While in our previous study, we focused only on CD138+ plasma cells, a marker of endometrial inflammation, in the stroma to evaluate endometrial immune status in women with PCOS and in RIF patients, the present study extended our examination to include both epithelial and stromal cell compartments with the aim of investigating epithelial-to-stromal proportions along the entire menstrual cycle, as well as in the context of these two infertility-associated conditions. IHC staining of the endometrial biopsies from controls (*n*=73) and women with PCOS (*n*=91) was performed at the University of Oulu, Oulu, Finland. The tissue samples from RIF patients (*n*=29) were processed at Tartu University Hospital, Tartu, Estonia. All of the stainings were performed following standard hematoxylin and eosin staining and IHC protocols.^14^^,^^18^ The stained slides were digitalized using a Leica SCN 400 Slide Scanner (Leica, Biosystems) and uploaded to Aiforia Hub (Aiforia Technologies Oy, Helsinki, Finland) (Fig. 1).

### Image analysis using the AI model

**Fig. 2:**
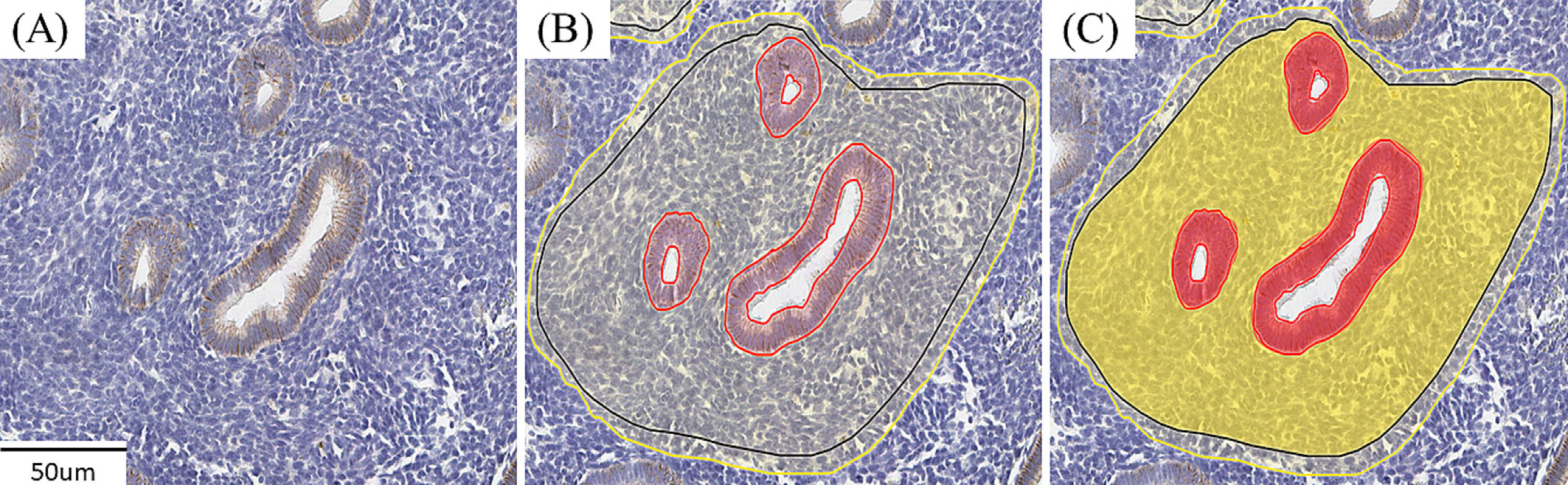
Examples of convolutional neural network (CNN)1 training. CNN1 training was performed within the area indicated by the solid black line. (A) Original image, (B) CNN1 training for segmenting the epithelium and stroma, and (C) the training result. The epithelium is marked red, and the stroma is marked yellow.

Our AI model, created on a supervised deep learning AI platform (Aiforia Technologies Oy, Helsinki, Finland), was structured by two individual convolutional neural networks (CNNs). The first CNN layer (CNN1), utilized in this study, was trained to segment both the epithelium and stroma using a total of 28.36 mm^2^ areas, including 2.56 mm^2^ of epithelium and 24.87 mm^2^ of stroma (Fig. 1, Fig. 2). Following the training step, validation was performed by two pathologists (OL and JK) at the University of Oulu, who were not involved in the AI model training. A total of 36 regions, including both the epithelium and stroma or only the stroma, from 18 whole-slide images were inspected. The validation set was selected considering the menstrual cycle phase (i.e., PE, SE) and the presence or absence of PCOS. The agreement between human annotations and the AI model detection was automatically computed on the same AI platform using precision, sensitivity, and F1 scores. A detailed description of the training and validation protocol for the second CNN layer (CNN2), which was trained to identify CD138+ plasma cells in the stroma, as well as the training and validation results for both CNNs, were documented in Lee et al.^14^

The AI-based analysis was performed for 193 whole-slide images. The areas of the epithelium and stroma were identified and calculated automatically by the AI model. The epithelium percentages were calculated manually by dividing the area of the epithelium [epithelium area (mm^2^)] by the total area [epithelium area (mm^2^) + stroma area (mm^2^)], then multiplying by 100.

A graphical overview of the methods and materials used in the study is presented in Fig. 1.

### Statistical analysis

Statistical analyses and graphic visualizations were conducted using IBM SPSS Statistics v28 (IBM Corp., Armonk, NY) and GraphPad Prism (version 9.3.0, GraphPad Software, San Diego, CA), respectively. For samples from multiple sample providers, both the epithelium percentages and clinical measurements were treated as individual samples. A linear mixed model was used to estimate the effects of the cycle phase and PCOS on epithelium percentages and to examine associations between the epithelium percentages and clinical features. The statistical differences in the epithelium percentages within the RIF group were analyzed using the Kruskal–Wallis test. Additionally, inter-pathologist variability was assessed using an intra-class correlation (ICC) estimate via a two-way mixed effects model with an absolute agreement model (ICC<0.5: poor; 0.5≤ICC<0.75: moderate; 0.75≤ICC<0.9: good; and ICC≥0.9: excellent reliability).^19^ A *p*-value for multiple testing was adjusted with the Bonferroni false-discovery rate (FDR) method, considering a significance level of *p*<0.05.

## Results

### The AI model performance and validation for CNN1

In our previous study, we demonstrated excellent performance of the AI model in identifying both tissue areas occupied by epithelial and stromal cells based on intensive training. Our AI model achieved high accuracy rates of 98.86% for CNN1 and 98.63% and 99.09% for the epithelium and stroma, respectively.^14^ Two experienced pathologists carried out CNN1 validation using 18 whole-slide images. The agreement between human annotations and the AI model performance showed F1 scores exceeding 82% for the epithelium and >96% for the stroma, signifying a consistent alignment in decisions between the pathologists and the AI model (Table 1). Moreover, inter-pathologist variability showed excellent reliability (ICC for the epithelium: 0.93, 95% CI, 0.87 to 0.97, *p*<0.001; ICC for the stroma: 0.93, 95% CI, 0.86 to 0.96, *p*<0.001).^14^ Hence, the AI model exhibited reliable and reproducible performance in delineating epithelial and stromal compartments.

**Table 1:** Results of the validation of convolutional neural network (CNN) 1 in 18 validation set.

%	Total (epithelium + stroma)		Epithelium		Stroma	
	Observer 1 vs. AI	Observer 2 vs. AI	Observer 1 vs. AI	Observer 2 vs. AI	Observer 1 vs. AI	Observer 2 vs. AI
Area error	5.92	5.57	7.66	8.24	4.18	2.90
Precision	95.69	96.85	85.13	91.32	99.12	98.65
Sensitivity	91.25	91.04	80.46	75.54	94.80	97.03
F1 score	93.42	93.85	82.73	82.69	96.91	97.83
Specificity	96.46	97.38	95.81	97.51	98.11	97.30
Accuracy	94.05	94.41	92.28	91.76	95.82	97.13

CNN1 consists of epithelium and stroma. The area error (false positives (FP) + false negatives (FN)), precision (true positives (TP)/[TP + FP]), sensitivity (TP/[TP + FN]), F1 score (2 × Precision × Sensitivity/[Precision + Sensitivity]), specificity (true negatives (TN)/[TN + FP]), and accuracy ([TP + TN]/[TP + TN + FP + FN]) percentages for CNN1 was calculated in comparison to each validator across all validation regions.

CNN, convolutional neural network.

### Healthy endometrium: Dynamic changes in endometrial epithelium proportions mirroring serum P4 levels

**Fig. 3:**
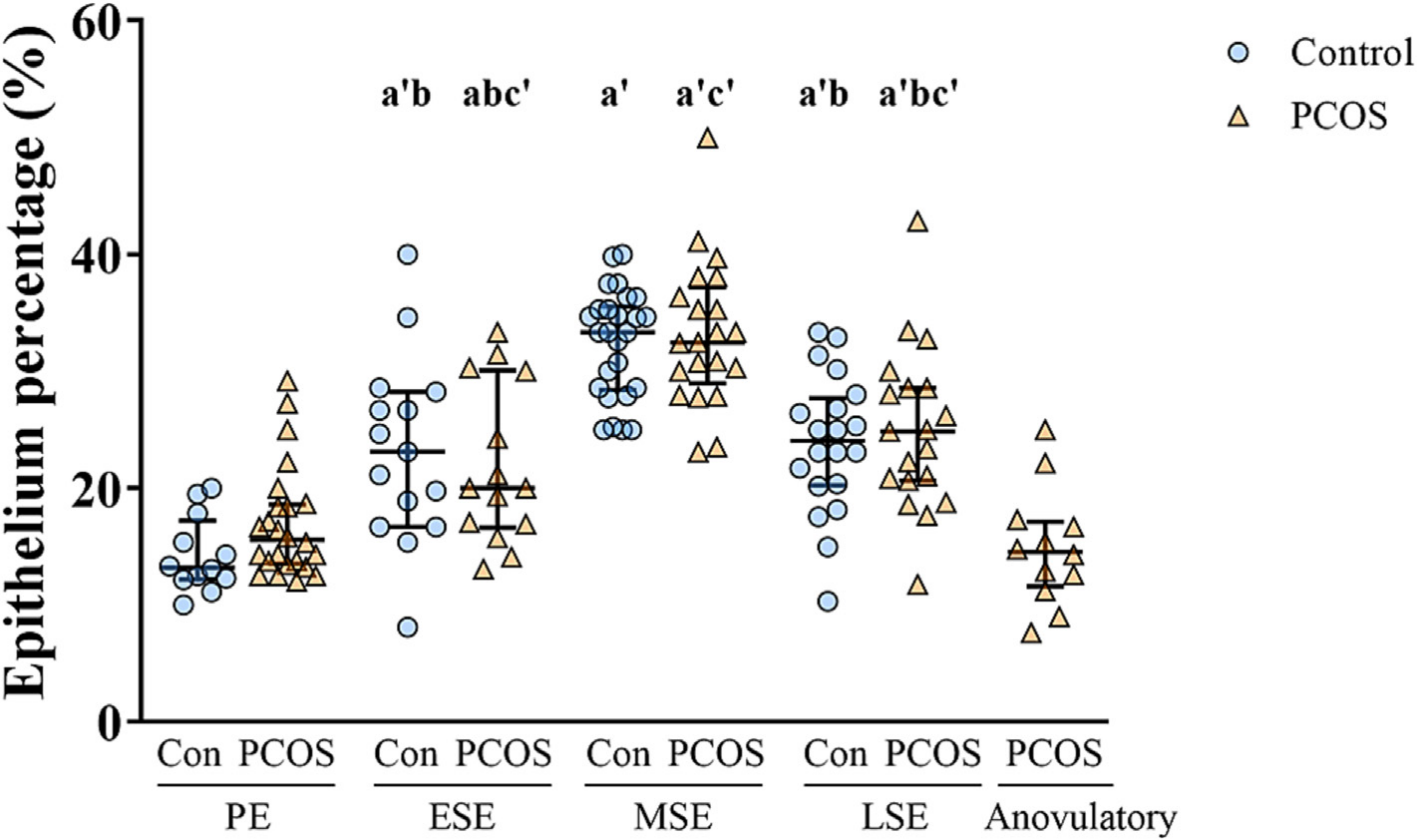
Epithelium percentages according to the cycle phases and PCOS diagnosis. Epithelium percentages in the controls (PE, *n*=12; ESE, *n*=15; MSE, *n*=26; LSE, *n*=20) and women with PCOS (PE, *n*=24; ESE, *n*=14; MSE, *n*=21; LSE, *n*=19; Anovulatory, *n*=12). Each symbol represents an individual sample in the controls (blue dot) and the PCOS samples (orange triangle), and the lines represent the upper quartiles, medians, and lower quartiles. ^a^*p*<0.05 and ^a′^*p*<0.001 when compared to the PE samples, ^b^*p*<0.001 when compared to the MSE samples, and ^c^*p*<0.01 and ^c′^*p*<0.001 when compared to the anovulatory samples by the mixed model analysis. PE, proliferative phase; ESE, early secretory phase; MSE, mid-secretory phase; LSE, late secretory phase.

We observed that the epithelium percentages, as quantified by the AI model, were following menstrual cycle phases (Fig. 3). The analysis revealed that the epithelium percentages increased from the PE toward the SE, peaking in the MSE and subsequently decreasing toward the LSE phase (PE, median 13.19%; ESE, median 23.10%; MSE, median 33.33%; LSE, median 24.04%). Next, we conducted correlation analyses between the epithelium percentages and other morphological and endocrine characteristics to validate whether the AI analysis results aligned with physiological changes during the menstrual cycle (Table 2). In the correlation analysis, we combined the three SEs, i.e., ESE, MSE, and LSE, into the SE for simplified comparison with higher statistical power. The epithelium percentages in the SE showed correlations with serum P4 level (*r*^2^ = 0.64, FDR<0.001) and FAI (*r*^2^ = -0.34, FDR=0.01).

**Table 2:** Correlations between the epithelium percentages and endometrial changes at different phases of menstrual cycle.

	Epithelium %	Endometrial thickness (mm)	P4 (nmol/L)	Testosterone (nmol/L)	FAI	FSH (IU/L)	LH (IU/L)	AMH (ng/ml)
*Control*								
Control PE (*n*=12)	13.19 [12.18,17.23]	5.25 [4.43,5.78] (0.41)	0.22 [0.19,0.31] (0.01)	1.01 [0.95,1.29] (0.23)	2.20 [1.50,2.76] (−0.52)	7.41 [6.68,8.75] (0.04)	7.69 [6.70,9.23] (0.09)	2.28 [1.22,4.64] (0.10)
Control SE (*n*=61)	27.73 [23.08,33.33]	9.85 [8.48,11.28] (−0.06)	31.00 [11.87,42.17] (0.64***)	1.04 [0.86,1.41] (0.03)	2.00 [1.24,2.93] (−0.34**)	3.62 [2.61,4.56] (−0.15)	7.47 [4.81,10.34] (0.04)	2.23 [1.35,4.38] (−0.06)

*PCOS*								
PCOS PE (*n*=24)	15.56 [13.52,18.60]	5.25 [4.40,5.95] (−0.07)	0.25 [0.19,0.30] (−0.04)	1.36 [1.08,1.70] (0.12)	2.74 [2.17,3.61] (−0.17)	6.82 [6.13,8.22] (−0.16)	8.72 [7.84,10.44] (−0.13)	5.45 [2.72,7.04] (0.07)
PCOS SE (*n*=54)	28.00 [20.78,32.92]	9.70 [8.12,10.65] (−0.18)	28.49 [10.07,44.71] (0.52***)	1.33 [1.00,1.72] (0.05)	2.38 [1.68,3.32] (−0.06)	3.73 [2.69,5.19] (−0.13)	8.95 [5.04,13.02] (0.10)	3.57 [2.34,5.80] (0.00)
PCOS anovulatory (*n*=12)	14.54 [11.57,17.13]	5.30 [5.00,7.40] (0.16)	0.34 [0.19,0.49] (0.07)	2.48 [1.96,3.20] (0.09)	7.67 [3.46,12.84] (−0.25)	6.33 [5.19,7.51] (0.09)	15.71 [10.68,20.02] (0.50)	8.59 [5.38,12.31] (0.15)

The epithelium percentages and clinical characteristics are presented as median with interquartile range [Q1;Q3]. The standardized coefficient was calculated by linear mixed effects models and are presented in brackets. *p* values were determined using log-transformed values of sex-hormone-related characteristics for the skewed data distribution. * *p*<0.05, ** *p*<0.01, and *** *p*<0.001.

PE, proliferative phase; SE, secretory phase; P4, progesterone; FAI, free androgen index; FSH, follicle-stimulating hormone; LH, luteinizing hormone; AMH, anti-Müllerian hormone.

### PCOS endometrium: Epithelium percentages and correlation with serum P4 levels

Concerning PCOS, the cycle phase analysis revealed results comparable to those observed in controls: The epithelium percentages increased from the PE toward the SE (PE, median 15.56%; ESE, median 20.00%; MSE, median 32.45%; LSE, median 24.18%) (Fig. 3). However, the anovulatory PCOS endometrium exhibited epithelium percentages similar to those in the PE endometrium (Anovulatory, median 14.54%, *p*=1.00). The epithelium percentages did not differ between the control and ovulatory PCOS samples in the same cycle phase (FDR_PE_=0.09, FDR_ESE_=0.67, FDR_MSE_=0.62, FDR_LSE_=0.58). Again, similar to the controls, the ovulatory PCOS endometrium in the SE exhibited a positive correlation with serum P4 level (*r*^2^ = 0.52, FDR<0.001) (Table 2). However, the anovulatory endometrium did not show any correlations with clinical characteristics, including sex hormone levels.

### RIF endometrium: Epithelium percentages in women with RIF

**Fig. 4:**
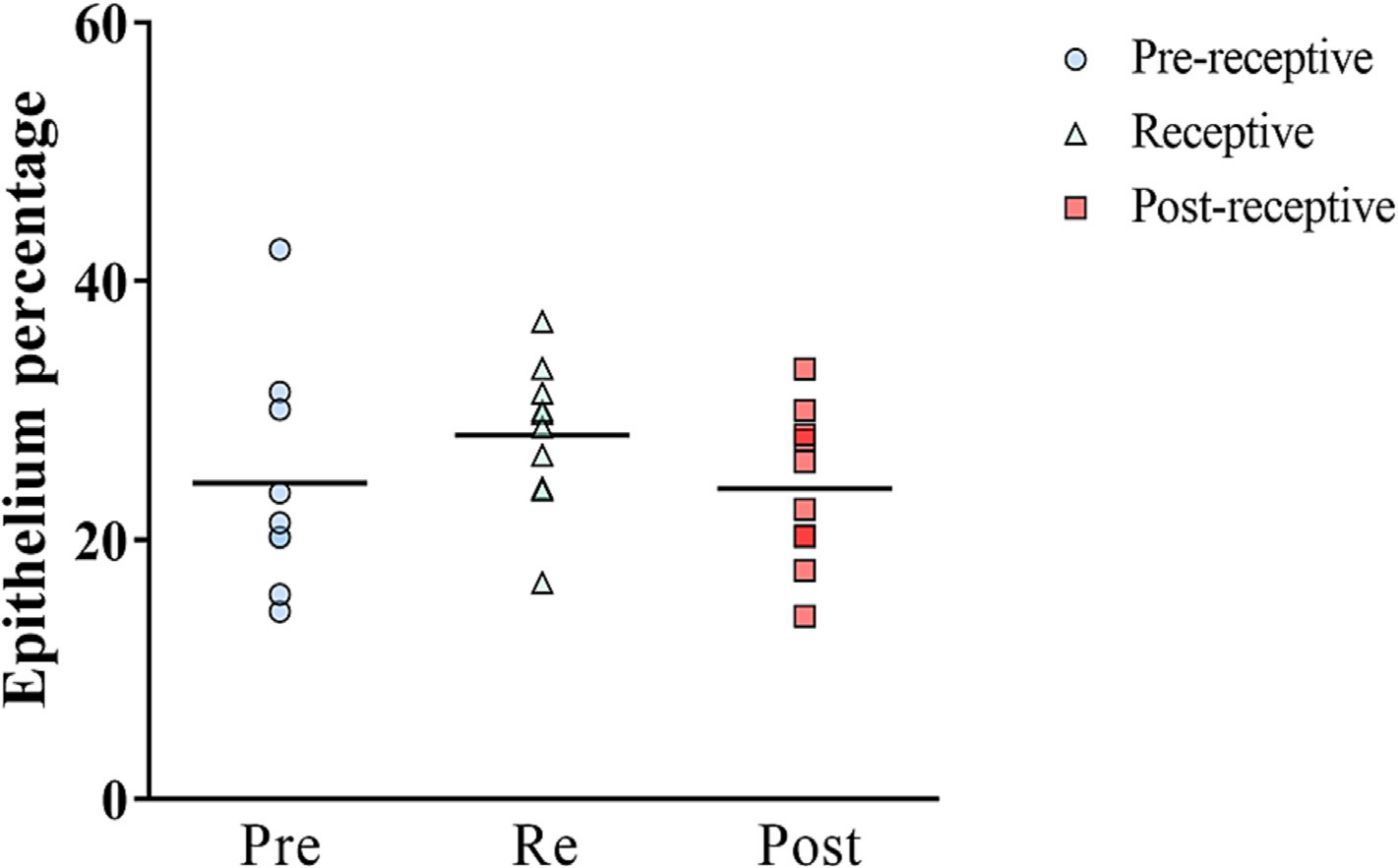
Epithelial proportion according to endometrial receptivity status in RIF patients. Epithelium percentages according to distinct endometrial receptivity profiles (Pre-receptive (Pre) RIF, *n*=9; Receptive (Re) RIF, *n*=10; Post-receptive (Post) RIF, *n*=10). Each symbol represents an individual sample in Pre (blue dot), Re (green triangle), and Post RIF (red square), and the lines represent the upper quartiles, medians, and lower quartiles. The statistical differences were calculated by the Kruskal–Wallis test. Pre, pre-receptive; Re, receptive; Post, post-receptive.

To investigate whether endometrial receptivity in response to the HRT is associated with epithelium percentages, we compared the percentages within the RIF samples, which were categorized into receptivity stages: pre-receptive, receptive, and post-receptive (Fig. 4). We revealed comparable epithelium percentages in the hormone-induced endometrium, irrespective of the receptivity test results (pre-receptive, 21.35%; receptive, 29.24%; post-receptive, 24.21%, FDR=0.88).

## Discussion

In this study, we evaluated the menstrual cycle phase-, PCOS-, and endometrial receptivity-dependent changes in endometrial epithelium-to-stromal ratios using an AI deep learning model. To our knowledge, this is the first study utilizing AI analysis in the assessment of the proportions of these two major endometrial compartments.

Previous protocols for the histological assessment of the endometrium employed additional staining with the p16 antibody to differentiate epithelial and stromal cell compartments by selectively staining only epithelial cells^20^^,^^21^ and using traditional image analysis software, which requires pre-processing steps, such as converting original images into grayscale or binary images for detecting staining intensity.^20^^,^^22^ These measurements were limited to specifically chosen tissue areas,^23^^,^^24^ and intra- and inter-observer variability was not considered.^23^ In contrast, here we showed that our AI model, which does not require additional staining or image conversion processes, analyzes a whole scanned slide within 1 min while producing highly accurate and reproducible results. The AI algorithm, the functionality and reliability of which were validated in our previous study,^14^ coincided with the pathologists’ assessment.

Previous studies highlight the advantages of digital pathology, which alleviates the workload of pathologists by facilitating rapid analysis, remote image assessments, and telepathology.^25^^,^^26^ Digitally stored slide images secure the viewing of fresh stains and preserve image integrity by eliminating risks that may degrade slide quality, such as breaking or losing of glass slides and exposure to adverse storage conditions.^26^ Moreover, the cloud-based platform we employed enhances the accessibility and convenience of image sharing among users, providing further opportunities for both research collaboration and education. AI has come to the forefront of digital pathology for pattern recognition and image classification,^27^^,^^28^ which can be utilized to predict the onset and progression of specific diseases in various clinical research fields.^29^, ^30^, ^31^, ^32^, ^33^, ^34^ AI technique is expected to disentangle current impediments in histopathological performance by analyzing massive amounts of whole-slide images within a short amount of time with a high pixel resolution,^35^ as demonstrated in this study. Additionally, the AI approach could also assist in bulk RNA sequencing analysis for deconvolution, where adjustment for the epithelial and stromal cellular ratio could be beneficial for identifying cell type composition-dependent and independent gene expression changes in the tissue.^13^

The AI analysis of the controls and ovulatory PCOS cases was in line with the endocrine changes within the menstrual cycle, indicating a positive correlation in the SE with the serum P4 value. Given that we did not observe any significant differences in epithelium percentages between control and ovulatory PCOS samples in any cycle phase, it is likely that epithelial development appears to be normal in women with PCOS upon achieving natural ovulation. Earlier studies have indicated that the anovulatory endometrium, which lacks P4 due to the absence of the corpus luteum, fails to undergo cyclic transformation,^9^^,^^36^ leading to abnormal development and secretion in the GE^3^ and inadequate stromal decidualization.^37^ Our results corroborate these previous findings by demonstrating similar epithelial percentages between anovulatory PCOS samples and ovulatory PCOS samples during PE. Overall, these findings bolster the reliability of the AI analysis.

The RIF endometrium displays structural, immunological, and genetic alterations,^8^ such as a thinner endometrium and reduced vascularity, compared to fertile women, affecting both epithelial cell growth and angiogenesis.^24^ In addition, alterations in the expression of cytokines^38^ and autoantibodies^39^ can make the endometrium hostile to embryo implantation in women with RIF. Furthermore, the dysregulated protein^40^^,^^41^ and transcriptomic profiles^37^^,^^42^ found in RIF patients involve aberrations in endometrial differentiation and cell adhesion processes, potentially leading to implantation failure. Here, we did not find significant histological variations within the hormone-induced endometrium in RIF patients, indicating the RIF endometrium showed normal epithelial and stromal development in response to HRT. Therefore, future studies should focus on molecular features of epithelial, stromal, and other cell types in the RIF endometrium and continue to explore targets related to infertility in order to better understand the endometrial dysfunction associated with RIF.

Besides employing a unique setup and AI analysis option, this study utilized a rare, large, and extremely well-characterized human endometrial sample set. We demonstrated that the AI algorithm, validated by two independent pathologists, has excellent accuracy in identifying endometrial morphological features dependent on the menstrual cycle, ovulatory status, and receptivity status, aligning with endometrial cyclic characteristics. However, the limited sample size from anovulatory PCOS women may hinder the exploration of correlations between epithelium percentages and clinical features. Similarly, the small sample size of RIF patients may result in an underestimation of the histological differences among different receptivity profiles. While the AI model demonstrated excellent accuracy in recognizing epithelium and stroma, a smaller training area of epithelium (2.56 mm^2^), compared to that of stroma (24.87 mm^2^), may lead to higher area training errors. This can be improved through more focused training on epithelium within larger regions of interest, leading to lower training errors. Despite these limitations, our study provides a solid foundation for AI-assisted endometrial histology investigations.

## Conclusion

The established AI algorithm rapidly and accurately identifies endometrial development by calculating areas occupied by epithelial and stromal cells. Our AI model demonstrates changes in epithelium percentages according to the menstrual cycle phase and the presence or absence of ovulation, mirroring endocrine changes. Overall, the AI model can potentially improve endometrial histology assessment by accelerating the analysis of the cellular composition of the tissue and by ensuring maximal objectivity for research and clinical purposes.

## Author contributions

T.T.P., A.S., R.K.A., and S.L. designed the study. T.T.P., A.S., R.K.A., E.K.K., and M.S. contributed to sample collection. K.K. and M.S. were involved in sample processing. O.L. and J.A.K. performed the validation. S.L. and E.K.K. performed image analysis. S.L. and R.K.A. performed the statistical analysis. All authors revised and approved the final version.

## Funding

This research was funded by the European Union’s Horizon 2020 research and innovation programme under the Marie Sklodowska-Curie grant agreement No. 813707 (MATER), The Academy of Finland, The Sigrid Jusélius Foundation, and 10.13039/501100009708Novo Nordisk Foundation. This research was also funded by the 10.13039/501100002301Estonian Research Council (grant PRG1076), Horizon 2020 innovation grant (ERIN, grant no. EU952516), 10.13039/501100006598Enterprise Estonia (grant no. EU48695), and MSCA-RISE-2020 project TRENDO (grant no. 101008193). The funders did not participate in any of the processes of the study.

## Declaration of competing interest

The authors declare that the research was conducted in the absence of any commercial or financial relationships that could be construed as a potential conflict of interest.
